# Supplementary material for: Serum TNF -α, IL-10 and IL-2 Trajectories and Outcomes in NSCLC and Melanoma Under Anti-PD-1 Therapy: Longitudinal Real-World Evidence from a Single Center
Source: Curr Issues Mol Biol. 2025 Sep 11;47(9):746. doi: 10.3390/cimb47090746 (PMC12468888; doi:10.3390/cimb47090746)
Supplement: Supplementary file 1 [file cimb-47-00746-s001.zip › Supplementary Materials-MixedModels-IL10.pdf]

Mixed Model Analysis

Notes

Output Created		12-JUN-2025 19:42:09
Comments		
Input	Active Dataset	DataSet1
	Filter	<none>
	Weight	<none>
	Split File	<none>
	N of Rows in Working Data File	174
Missing Value Handling	Definition of Missing	User-defined missing values are treated as missing.
	Cases Used	Statistics are based on all cases with valid data for all variables in the model.
Syntax		MIXED IL10 BY Type Time<br>/CRITERIA=DFMETHOD (SATTERTHWAITE) CIN (95) MXITER(100) MXSTEP(10) SCORING(1)<br>SINGULAR (0.000000000001) HCONVERGE (0.00000001, RELATIVE) LCONVERGE(0, ABSOLUTE) PCONVERGE (0,<br>ABSOLUTE)<br>/FIXED=Type Time Type*Time | SSTYPE(3)<br>/METHOD=REML<br>/RANDOM=Time | COVTYPE(VC)<br>/RANDOM=Type | COVTYPE(VC)<br>/REPEATED=Time | SUBJECT(id) COVTYPE (AR1)<br>/EMMEANS=TABLES (Time) COMPARE ADJ (BONFERRONI)<br>/EMMEANS=TABLES (Type) COMPARE ADJ (BONFERRONI)<br>/EMMEANS=TABLES (Type*Time) .
Resources	Processor Time	00:00:00.04
	Elapsed Time	00:00:00.00

Warnings

The final Hessian matrix is not positive definite although all convergence criteria are satisfied. The MIXED procedure continues despite this warning. Validity of subsequent results cannot be ascertained.

Model Dimension[a]

		Number of Levels	Covariance Structure	Number of Parameters	Subject Variables
Fixed Effects	Intercept	1		1	
	Type	2		1	
	Time	3		2	
	Type * Time	6		2	
Random Effects	Time	3	Variance Components	1	
	Type	2	Variance Components	1	
Repeated Effects	Time	3	First-Order Autoregressive	2	id
Total		20		10	

Model Dimension[a]

		Number of Subjects
Fixed Effects	Intercept	
	Type	
	Time	
	Type * Time	
Random Effects	Time	
	Type	
Repeated Effects	Time	58
Total		

a. Dependent Variable: IL10 [pg/ml].

Information Criteria[a]

-2 Restricted Log Likelihood	1504.63752
Akaike's Information Criterion (AIC)	1512.63752
Hurvich and Tsai's Criterion (AICC)	1513.08696
Bozdogan's Criterion (CAIC)	1526.81070
Schwarz's Bayesian Criterion (BIC)	1522.81070

The information criteria are displayed in smaller-is-better form.

a. Dependent Variable: IL10 [pg/ml].

Fixed Effects

Type III Tests of Fixed Effects[a]

Source	Numerator df	Denominator df	F	Sig.
Intercept	1	73.922	8.450	.005
Type	1	73.922	2.434	.123
Time	2	58.196	4.670	.013
Type * Time	2	58.196	2.870	.065

a. Dependent Variable: IL10 [pg/ml].

Covariance Parameters

Estimates of Covariance Parameters[a]

Parameter		Estimate	Std. Error
Repeated Measures	AR1 diagonal	559848.029	89352.288
	AR1 rho	.658	.069
Time	Variance	.000[b]	.000
Type	Variance	.000[b]	.000

a. Dependent Variable: IL10 [pg/ml].

b. This covariance parameter is redundant.

Estimated Marginal Means

1. Time

Estimates[a]

Time	Mean	Std. Error	df	95% Confidence Interval Lower Bound	95% Confidence Interval Upper Bound
1	97.224	112.186	78.516	-126.098	320.547
2	492.239	144.053	93.987	206.217	778.261
3	421.368	181.076	93.436	61.810	780.926

a. Dependent Variable: IL10 [pg/ml].

Pairwise Comparisons[a]

(I) Time	(J) Time	Mean Difference (I-J)	Std. Error	df	Sig.[c]	95% Confidence ...[c] Lower Bound
1	2	-395.015*	129.497	57.274	.010	-714.397
	3	-324.144	185.660	74.782	.255	-778.829
2	1	395.015*	129.497	57.274	.010	75.632
	3	70.871	161.935	54.642	1.000	-329.096
3	1	324.144	185.660	74.782	.255	-130.541
	2	-70.871	161.935	54.642	1.000	-470.837

Pairwise Comparisons[a]

(I) Time	(J) Time	95% Confidence Interval for ...[c] Upper Bound
1	2	-75.632
	3	130.541
2	1	714.397
	3	470.837
3	1	778.829
	2	329.096

Based on estimated marginal means

*. The mean difference is significant at the .05 level.

a. Dependent Variable: IL10 [pg/ml].

c. Adjustment for multiple comparisons: Bonferroni.

Univariate Tests[a]

Numerator df	Denominator df	F	Sig.
2	56.275	4.670	.013

The F tests the effect of Time. This test is based on the linearly independent pairwise comparisons among the estimated marginal means.

a. Dependent Variable: IL10 [pg/ml].

2. Type

Estimates[a]

Type	Mean	Std. Error	df	95% Confidence Interval Lower Bound	95% Confidence Interval Upper Bound
Melanoma	517.765	197.584	72.464	123.932	911.598
NSCLC	156.123	121.245	77.732	-85.271	397.517

a. Dependent Variable: IL10 [pg/ml].

Pairwise Comparisons[a]

(I) Type	(J) Type	Mean Difference (I-J)	Std. Error	df	Sig.[b]	95% Confidence ...[b] Lower Bound
Melanoma	NSCLC	361.642	231.818	73.922	.123	-100.274
NSCLC	Melanoma	-361.642	231.818	73.922	.123	-823.558

Pairwise Comparisons[a]

(I) Type	(J) Type	95% Confidence Interval for ...[b] Upper Bound
Melanoma	NSCLC	823.558
NSCLC	Melanoma	100.274

Based on estimated marginal means

a. Dependent Variable: IL10 [pg/ml].

b. Adjustment for multiple comparisons: Bonferroni.

Univariate Tests[a]

Numerator df	Denominator df	F	Sig.
1	73.922	2.434	.123

The F tests the effect of Type. This test is based on the linearly independent pairwise comparisons among the estimated marginal means.

a. Dependent Variable: IL10 [pg/ml].

3. Type * Time[a]

Type	Time	Mean	Std. Error	df	95% Confidence Interval Lower Bound	95% Confidence Interval Upper Bound
Melanoma	1	79.193	193.192	78.516	-305.383	463.770
	2	780.299	247.991	93.986	287.907	1272.692
	3	693.802	300.175	93.902	97.788	1289.815
NSCLC	1	115.255	114.104	78.516	-111.885	342.396
	2	204.179	146.649	93.989	-86.998	495.355
	3	148.935	202.603	91.136	-253.504	551.374

a. Dependent Variable: IL10 [pg/ml].
