# Supplementary material for: Serum TNF -α, IL-10 and IL-2 Trajectories and Outcomes in NSCLC and Melanoma Under Anti-PD-1 Therapy: Longitudinal Real-World Evidence from a Single Center
Source: Curr Issues Mol Biol. 2025 Sep 11;47(9):746. doi: 10.3390/cimb47090746 (PMC12468888; doi:10.3390/cimb47090746)
Supplement: Supplementary file 1 [file cimb-47-00746-s001.zip › Supplementary Materials-MixedModels-IL2.pdf]

Mixed Model Analysis

Notes

Output Created		12-JUN-2025 19:38:44
Comments		
Input	Active Dataset	DataSet1
	Filter	<none>
	Weight	<none>
	Split File	<none>
	N of Rows in Working Data File	174
Missing Value Handling	Definition of Missing	User-defined missing values are treated as missing.
	Cases Used	Statistics are based on all cases with valid data for all variables in the model.
Syntax		MIXED IL2 BY Type Time /CRITERIA=DFMETHOD (SATTERTHWAITE) CIN (95) MXITER(100) MXSTEP(10) SCORING(1) SINGULAR (0.000000000001) HCONVERGE (0.00000001, RELATIVE) LCONVERGE(0, ABSOLUTE) PCONVERGE (0, ABSOLUTE) /FIXED=Type Time Type*Time | SSTYPE(3) /METHOD=REML /RANDOM=Time | COVTYPE(VC) /RANDOM=Type | COVTYPE(VC) /REPEATED=Time | SUBJECT(id) COVTYPE (AR1) /EMMEANS=TABLES (Time) COMPARE ADJ (BONFERRONI) /EMMEANS=TABLES (Type) COMPARE ADJ (BONFERRONI) /EMMEANS=TABLES (Type*Time) .
Resources	Processor Time	00:00:00.05
	Elapsed Time	00:00:00.00

Warnings

The final Hessian matrix is not positive definite although all convergence criteria are satisfied. The MIXED procedure continues despite this warning. Validity of subsequent results cannot be ascertained.

Model Dimension[a]

		Number of Levels	Covariance Structure	Number of Parameters	Subject Variables
Fixed Effects	Intercept	1		1	
	Type	2		1	
	Time	3		2	
	Type * Time	6		2	
Random Effects	Time	3	Variance Components	1	
	Type	2	Variance Components	1	
Repeated Effects	Time	3	First-Order Autoregressive	2	id
Total		20		10	

Model Dimension[a]

		Number of Subjects
Fixed Effects	Intercept	
	Type	
	Time	
	Type * Time	
Random Effects	Time	
	Type	
Repeated Effects	Time	58
Total		

a. Dependent Variable: IL2 [pg/ml].

Information Criteria[a]

-2 Restricted Log Likelihood	1422.96736
Akaike's Information Criterion (AIC)	1430.96736
Hurvich and Tsai's Criterion (AICC)	1431.41679
Bozdogan's Criterion (CAIC)	1445.14053
Schwarz's Bayesian Criterion (BIC)	1441.14053

The information criteria are displayed in smaller-is-better form.

a. Dependent Variable: IL2 [pg/ml].

Fixed Effects

Type III Tests of Fixed Effects[a]

Source	Numerator df	Denominator df	F	Sig.
Intercept	1	55.683	1.400	.242
Type	1	55.683	.175	.678
Time	2	74.343	.620	.541
Type * Time	2	74.343	.374	.689

a. Dependent Variable: IL2 [pg/ml].

Covariance Parameters

Estimates of Covariance Parameters[a]

Parameter		Estimate	Std. Error
Repeated Measures	AR1 diagonal	188986.199	27793.951
	AR1 rho	.174	.130
Time	Variance	.000[b]	.000
Type	Variance	.000[b]	.000

a. Dependent Variable: IL2 [pg/ml].

b. This covariance parameter is redundant.

Estimated Marginal Means

1. Time

Estimates[a]

Time	Mean	Std. Error	df	95% Confidence Interval Lower Bound	95% Confidence Interval Upper Bound
1	12.825	65.181	92.468	-116.621	142.271
2	133.469	94.685	93.861	-54.534	321.473
3	65.573	118.394	93.991	-169.501	300.646

a. Dependent Variable: IL2 [pg/ml].

Pairwise Comparisons[a]

(I) Time	(J) Time	Mean Difference (I-J)	Std. Error	df	Sig.[b]	95% Confidence ...[b] Lower Bound
1	2	-120.644	108.347	66.466	.809	-386.753
	3	-52.747	134.200	92.901	1.000	-379.940
2	1	120.644	108.347	66.466	.809	-145.464
	3	67.897	140.962	56.913	1.000	-279.828
3	1	52.747	134.200	92.901	1.000	-274.446
	2	-67.897	140.962	56.913	1.000	-415.622

Pairwise Comparisons[a]

(I) Time	(J) Time	95% Confidence Interval for ...[b] Upper Bound
1	2	145.464
	3	274.446
2	1	386.753
	3	415.622
3	1	379.940
	2	279.828

Based on estimated marginal means

a. Dependent Variable: IL2 [pg/ml].

b. Adjustment for multiple comparisons: Bonferroni.

Univariate Tests[a]

Numerator df	Denominator df	F	Sig.
2	62.486	.620	.541

The F tests the effect of Time. This test is based on the linearly independent pairwise comparisons among the estimated marginal means.

a. Dependent Variable: IL2 [pg/ml].

2. Type

Estimates[a]

Type	Mean	Std. Error	df	95% Confidence Interval Lower Bound	95% Confidence Interval Upper Bound
Melanoma	45.676	100.541	53.218	-155.964	247.316
NSCLC	95.569	64.394	62.040	-33.151	224.289

a. Dependent Variable: IL2 [pg/ml].

Pairwise Comparisons[a]

(I) Type	(J) Type	Mean Difference (I-J)	Std. Error	df	Sig.[b]	95% Confidence ...[b] Lower Bound
Melanoma	NSCLC	-49.893	119.394	55.683	.678	-289.099
NSCLC	Melanoma	49.893	119.394	55.683	.678	-189.313

Pairwise Comparisons[a]

(I) Type	(J) Type	95% Confidence Interval for ...[b] Upper Bound
Melanoma	NSCLC	189.313
NSCLC	Melanoma	289.099

Based on estimated marginal means

a. Dependent Variable: IL2 [pg/ml].

b. Adjustment for multiple comparisons: Bonferroni.

Univariate Tests[a]

Numerator df	Denominator df	F	Sig.
1	55.683	.175	.678

The F tests the effect of Type. This test is based on the linearly independent pairwise comparisons among the estimated marginal means.

a. Dependent Variable: IL2 [pg/ml].

3. Type * Time[a]

Type	Time	Mean	Std. Error	df	95% Confidence Interval	
					Lower Bound	Upper Bound
Melanoma	1	8.273	112.246	92.468	-214.642	231.187
	2	47.652	162.985	93.862	-275.966	371.269
	3	81.103	193.543	93.903	-303.186	465.392
NSCLC	1	17.378	66.295	92.468	-114.281	149.036
	2	219.287	96.421	93.858	27.837	410.738
	3	50.042	136.416	93.899	-220.819	320.903

a. Dependent Variable: IL2 [pg/ml].
