# Supplementary material for: Serum TNF -α, IL-10 and IL-2 Trajectories and Outcomes in NSCLC and Melanoma Under Anti-PD-1 Therapy: Longitudinal Real-World Evidence from a Single Center
Source: Curr Issues Mol Biol. 2025 Sep 11;47(9):746. doi: 10.3390/cimb47090746 (PMC12468888; doi:10.3390/cimb47090746)
Supplement: Supplementary file 1 [file cimb-47-00746-s001.zip › Supplementary Materials-MixedModels-TNFalpha.pdf]

Mixed Model Analysis

Notes

Output Created		12-JUN-2025 19:31:57
Comments		
Input	Active Dataset	DataSet1
	Filter	<none>
	Weight	<none>
	Split File	<none>
	N of Rows in Working Data File	174
Missing Value Handling	Definition of Missing	User-defined missing values are treated as missing.
	Cases Used	Statistics are based on all cases with valid data for all variables in the model.
Syntax		MIXED TNFalpha BY Type Time<br>/CRITERIA=DFMETHOD (SATTERTHWAITE) CIN (95) MXITER(100) MXSTEP(10) SCORING(1)<br>SINGULAR (0.000000000001) HCONVERGE (0.00000001, RELATIVE) LCONVERGE(0, ABSOLUTE) PCONVERGE (0,<br>ABSOLUTE)<br>/FIXED=Type Time Type*Time | SSTYPE(3)<br>/METHOD=REML<br>/RANDOM=Time | COVTYPE(VC)<br>/RANDOM=Type | COVTYPE(VC)<br>/REPEATED=Time | SUBJECT(id) COVTYPE (AR1)<br>/EMMEANS=TABLES (Time) COMPARE ADJ (BONFERRONI)<br>/EMMEANS=TABLES (Type) COMPARE ADJ (BONFERRONI)<br>/EMMEANS=TABLES (Type*Time) .
Resources	Processor Time	00:00:00.04
	Elapsed Time	00:00:00.00

Warnings

The final Hessian matrix is not positive definite although all convergence criteria are satisfied. The MIXED procedure continues despite this warning. Validity of subsequent results cannot be ascertained.

Model Dimension[a]

		Number of Levels	Covariance Structure	Number of Parameters	Subject Variables
Fixed Effects	Intercept	1		1	
	Type	2		1	
	Time	3		2	
	Type * Time	6		2	
Random Effects	Time	3	Variance Components	1	
	Type	2	Variance Components	1	
Repeated Effects	Time	3	First-Order Autoregressive	2	id
Total		20		10	

Model Dimension[a]

		Number of Subjects
Fixed Effects	Intercept	
	Type	
	Time	
	Type * Time	
Random Effects	Time	
	Type	
Repeated Effects	Time	58
Total		

a. Dependent Variable: TNF [pg/ml].

Information Criteria[a]

-2 Restricted Log Likelihood	1264.78767
Akaike's Information Criterion (AIC)	1272.78767
Hurvich and Tsai's Criterion (AICC)	1273.23711
Bozdogan's Criterion (CAIC)	1286.96085
Schwarz's Bayesian Criterion (BIC)	1282.96085

The information criteria are displayed in smaller-is-better form.

a. Dependent Variable: TNF [pg/ml].

Fixed Effects

Type III Tests of Fixed Effects[a]

Source	Numerator df	Denominator df	F	Sig.
Intercept	1	70.291	36.686	<.001
Type	1	70.291	3.812	.055
Time	2	47.137	3.055	.057
Type * Time	2	47.137	.985	.381

a. Dependent Variable: TNF [pg/ml].

Covariance Parameters

Estimates of Covariance Parameters[a]

Parameter		Estimate	Std. Error
Repeated Measures	AR1 diagonal	54052.632	9294.045
	AR1 rho	.816	.046
Time	Variance	.000[b]	.000
Type	Variance	.000	3.887E+11

a. Dependent Variable: TNF [pg/ml].

b. This covariance parameter is redundant.

Estimated Marginal Means

1. Time

Estimates[a]

Time	Mean	Std. Error	df	95% Confidence Interval Lower Bound	95% Confidence Interval Upper Bound
1	168.599	34.859	67.648	99.032	238.165
2	242.592	40.983	90.481	161.178	324.006
3	244.272	49.724	93.614	145.539	343.005

a. Dependent Variable: TNF [pg/ml].

Pairwise Comparisons[a]

(I) Time	(J) Time	Mean Difference (I-J)	Std. Error	df	Sig.[b]	95% Confidence ...[b] Lower Bound
1	2	-73.993	30.188	46.764	.054	-148.954
	3	-75.674	45.484	57.092	.305	-187.863
2	1	73.993	30.188	46.764	.054	-.968
	3	-1.681	37.556	46.388	1.000	-94.966
3	1	75.674	45.484	57.092	.305	-36.516
	2	1.681	37.556	46.388	1.000	-91.605

Pairwise Comparisons[a]

(I) Time	(J) Time	95% Confidence Interval for ...[b] Upper Bound
1	2	.968
	3	36.516
2	1	148.954
	3	91.605
3	1	187.863
	2	94.966

Based on estimated marginal means

a. Dependent Variable: TNF [pg/ml].

b. Adjustment for multiple comparisons: Bonferroni.

Univariate Tests[a]

Numerator df	Denominator df	F	Sig.
2	46.678	3.055	.057

The F tests the effect of Time. This test is based on the linearly independent pairwise comparisons among the estimated marginal means.

a. Dependent Variable: TNF [pg/ml].

2. Type

Estimates[a]

Type	Mean	Std. Error	df	95% Confidence Interval Lower Bound	95% Confidence Interval Upper Bound
Melanoma	288.918	61.750	69.196	165.737	412.099
NSCLC	148.057	37.308	73.286	73.708	222.406

a. Dependent Variable: TNF [pg/ml].

Pairwise Comparisons[a]

(I) Type	(J) Type	Mean Difference (I-J)	Std. Error	df	Sig.[b]	95% Confidence ...[b] Lower Bound
Melanoma	NSCLC	140.861	72.145	70.291	.055	-3.017
NSCLC	Melanoma	-140.861	72.145	70.291	.055	-284.739

Pairwise Comparisons[a]

(I) Type	(J) Type	95% Confidence Interval for ...[b] Upper Bound
Melanoma	NSCLC	284.739
NSCLC	Melanoma	3.017

Based on estimated marginal means

a. Dependent Variable: TNF [pg/ml].

b. Adjustment for multiple comparisons: Bonferroni.

Univariate Tests[a]

Numerator df	Denominator df	F	Sig.
1	70.291	3.812	.055

The F tests the effect of Type. This test is based on the linearly independent pairwise comparisons among the estimated marginal means.

a. Dependent Variable: TNF [pg/ml].

3. Type * Time[a]

Type	Time	Mean	Std. Error	df	95% Confidence Interval Lower Bound	95% Confidence Interval Upper Bound
Melanoma	1	211.545	60.029	67.648	91.747	331.343
	2	327.804	70.560	90.464	187.635	467.973
	3	327.405	83.223	93.980	162.164	492.647
NSCLC	1	125.652	35.455	67.648	54.897	196.408
	2	157.379	41.710	90.530	74.521	240.237
	3	161.139	54.440	91.018	53.001	269.277

a. Dependent Variable: TNF [pg/ml].
