# Supplementary material for: Serum TNF -α, IL-10 and IL-2 Trajectories and Outcomes in NSCLC and Melanoma Under Anti-PD-1 Therapy: Longitudinal Real-World Evidence from a Single Center
Source: Curr Issues Mol Biol. 2025 Sep 11;47(9):746. doi: 10.3390/cimb47090746 (PMC12468888; doi:10.3390/cimb47090746)
Supplement: Supplementary file 1 [file cimb-47-00746-s001.zip › Supplementary Materials-Statistical analysis - Correlations and Covariate analysis.pdf]

Supplemental materials

Cox proportional hazards models results

LOG1P+Z: Time-updated Cox (joint)

Cytokines (joint, z, log1p)	N subjects	N intervals	HR	CI_low	CI_high	p
**TNFa (z, log1p)**	**44**	**99**	**4.40948**	**1.14481**	**16.98406**	**0.03104**
**IL2 (z, log1p)**	**44**	**99**	**0.526805**	**0.356484**	**0.778501**	**0.001297**
IL10 (z, log1p)	44	99	0.524063	0.099461	2.761297	0.446019

LOG1P+Z: Time-updated Cox (single)

Cytokine (time-varying, z, log1p)	N subjects	N intervals	HR	CI_low	CI_high	p
TNFa (z, log1p)	44	99	1.442847	0.92188	2.258221	0.108691
IL2 (z, log1p)	44	99	0.974434	0.730322	1.300142	0.860275
IL10 (z, log1p)	44	99	1.26245	0.747522	2.132087	0.383398

LOG1P+Z: Static Cox (single)

timepoint	cytokine	predictor	N	HR	CI_low	CI_high	p
baseline	TNFA	at baseline - tnfa_log1p_z	44	1.365795	0.892602	2.089842	0.150869
baseline	IL2	at baseline - il2_log1p_z	44	0.970436	0.651903	1.444611	0.882467
baseline	IL10	at baseline - il10_log1p_z	44	1.122011	0.71121	1.770094	0.620655
3m	TNFA	at 3m - tnfa_log1p_z	21				
3m	IL10	at 3m - il10_log1p_z	21	0.257212	0	inf	0.999999

Fixed – Cox proportional hazard model

timepoint	cytokine_column	N	HR	CI_low	CI_high	p
**baseline**	**at baseline - tnfa**	**44**	**1.0109608**	**0.99988217**	**1.02216219**	**0.05249655**
baseline	at baseline - il2	44	0.99406236	0.94649164	1.04402399	0.81185789
baseline	at baseline - il10	44	0.99773771	0.98998533	1.00555079	0.5692912

Data attrition summary

Timepoint	N with cytokine	N total	Percent with	Percent missing
3 months	27	58	46.6	53.4
6 months	15	58	25.9	74.1

Comparisons Baseline vs 3m

Variable	Level/Statistic	3m: With follow-up	3m: Without follow-up	p
type	0	20 (74.1%)	23 (74.2%)	1
type	1	7 (25.9%)	8 (25.8%)	1
smokers	0	8 (29.6%)	9 (29.0%)	1
smokers	1	19 (70.4%)	22 (71.0%)	1
pd-l1	0.0	9 (42.9%)	13 (56.5%)	0.546694759
pd-l1	1.0	12 (57.1%)	10 (43.5%)	0.546694759
corticotherapy	0	20 (74.1%)	17 (54.8%)	0.173606652
corticotherapy	1	7 (25.9%)	14 (45.2%)	0.173606652
antibiotics >6 days	0	20 (74.1%)	24 (77.4%)	1
antibiotics >6 days	1	7 (25.9%)	7 (22.6%)	1
type of nsclc	adenocarcinoma	10 (50.0%)	15 (65.2%)	0.365200018
type of nsclc	squamous cell carcinoma	10 (50.0%)	8 (34.8%)	0.365200018
braf status	0.0	2 (28.6%)	5 (62.5%)	0.314685315
braf status	1.0	5 (71.4%)	3 (37.5%)	0.314685315
ecog	median [IQR]	0.00 [0.00–1.00]	1.00 [0.00–1.00]	0.233934896
line of nivolumab	median [IQR]	2.00 [2.00–2.00]	2.00 [1.00–2.00]	0.476242118
at baseline - TNFa	median [IQR]	89.55 [63.23–122.95]	85.95 [78.97–109.50]	0.558797599
at baseline - il2	median [IQR]	3.85 [3.20–4.53]	3.90 [3.52–4.22]	0.937854105
at baseline - il10	median [IQR]	7.95 [6.45–14.82]	5.96 [4.71–11.22]	0.030847969

Comparisons Baseline vs 6m

Variable	Level/Statistic	6m: With follow-up	6m: Without follow-up	p
type	0	10 (66.7%)	33 (76.7%)	0.501536958
type	1	5 (33.3%)	10 (23.3%)	0.501536958
smokers	0	6 (40.0%)	11 (25.6%)	0.33357251
smokers	1	9 (60.0%)	32 (74.4%)	0.33357251
pd-l1	0.0	3 (27.3%)	19 (57.6%)	0.162277728
pd-l1	1.0	8 (72.7%)	14 (42.4%)	0.162277728
corticotherapy	0	12 (80.0%)	25 (58.1%)	0.211993037
corticotherapy	1	3 (20.0%)	18 (41.9%)	0.211993037
antibiotics >6 days	0	12 (80.0%)	32 (74.4%)	1
antibiotics >6 days	1	3 (20.0%)	11 (25.6%)	1
type of nsclc	adenocarcinoma	5 (50.0%)	20 (60.6%)	0.717354525

type of nsclc	squamous cell carcinoma	5 (50.0%)	13 (39.4%)	0.717354525
braf status	0.0	2 (40.0%)	5 (50.0%)	1
braf status	1.0	3 (60.0%)	5 (50.0%)	1
ecog	median [IQR]	0.00 [0.00–1.00]	0.00 [0.00–1.00]	0.332083075
line of nivolumab	median [IQR]	2.00 [2.00–2.00]	2.00 [2.00–2.00]	0.825556392
at baseline - tnfa	median [IQR]	89.55 [70.28–110.15]	85.95 [76.50–116.72]	0.866020382
at baseline - il2	median [IQR]	3.85 [3.25–4.30]	3.90 [3.48–4.25]	0.769449223
at baseline - il10	median [IQR]	7.55 [5.80–10.35]	7.65 [5.28–14.34]	0.824334384

Spearman vs Survival: NSCLC Adenocarcinoma

Subtype	ytokine (timepoint)	Column	N	rho	_low	CI_high	p	q_BH
CLC - Adenocarcinoma	IL10 (baseline)	aseline - il10	25	.34385	55059	).059348	9238	0.484569
CLC - Adenocarcinoma	TNFa (baseline)	aseline - tnfa	25	.32462	53792	).080891	13376	0.484569
CLC - Adenocarcinoma	TNFa (3m)	t 3m - tnfa	10	478788	21593	).851671	51523	0.484569
CLC - Adenocarcinoma	IL2 (3m)	it 3m - il2	10	236364	46202	).753808	10885	0.802077
CLC - Adenocarcinoma	IL10 (3m)	t 3m - il10	10	-0.2	73684	).491521	79584	0.802077
CLC - Adenocarcinoma	TNFa (6m)	t 6m - tnfa	5	0.3	79185	).934834	23838	0.802077
CLC - Adenocarcinoma	IL2 (6m)	it 6m - il2	5	-0.3	93483	).791854	23838	0.802077
CLC - Adenocarcinoma	IL2 (baseline)	baseline - il2	25	.06387	4477	).339837	51651	0.856857
CLC - Adenocarcinoma	IL10 (6m)	t 6m - il10	5	0.1	35796	0.90263	72889	0.872889

Spearman vs Survival: NSCLC Squamous

Subtype	Cytokine (timepoint)	Column	N	rho	CI_low	CI_high	p	q_BH
NSCLC - Squamous	IL10 (3m)	at 3m - il10	10	-0.70909	-0.92551	-0.14356	0.021666	0.168237
NSCLC - Squamous	IL2 (6m)	at 6m - il2	5	0.9	0.086102	0.993438	0.037386	0.168237
NSCLC - Squamous	TNFa (baseline)	at baseline - tnfa	18	-0.35191	-0.70324	0.137562	0.152107	0.456321
NSCLC - Squamous	IL10 (baseline)	at baseline - il10	18	0.199174	-0.29514	0.609379	0.428158	0.701817
NSCLC - Squamous	IL2 (3m)	at 3m - il2	10	-0.27273	-0.77011	0.43089	0.445838	0.701817
NSCLC - Squamous	IL2 (baseline)	at baseline - il2	18	0.147575	-0.34292	0.574839	0.55897	0.701817
NSCLC - Squamous	TNFa (3m)	at 3m - tnfa	10	-0.2	-0.73684	0.491521	0.579584	0.701817
NSCLC - Squamous	TNFa (6m)	at 6m - tnfa	5	0.3	-0.79185	0.934834	0.623838	0.701817
NSCLC - Squamous	IL10 (6m)	at 6m - il10	5	0	-0.88227	0.882266	1	1

Spearman overall

Spearman correlation								
Variable 1	Variable 2	n	rho	CI_low	CI_high	p	q_BH	matrix
at baseline - tnfa	at baseline - il2	43	0.54	0.29	0.73	0.0002	0.0005	NSCLC - Baseline
at baseline - tnfa	at baseline - il10	43	0.39	0.10	0.61	0.0106	0.0159	NSCLC - Baseline
at baseline - il2	at baseline - il10	43	0.15	-0.16	0.43	0.3340	0.3340	NSCLC - Baseline
at 3m - tnfa	at 3m - il2	20	0.78	0.52	0.91	0.0000	0.0001	NSCLC - 3 months
at 3m - tnfa	at 3m - il10	20	0.60	0.21	0.82	0.0051	0.0077	NSCLC - 3 months
at 3m - il2	at 3m - il10	20	0.49	0.06	0.76	0.0298	0.0298	NSCLC - 3 months
at 6m - tnfa	at 6m - il2	10	0.15	-0.53	0.71	0.6876	0.7635	NSCLC - 6 months
at 6m - tnfa	at 6m - il10	10	0.55	-0.12	0.88	0.0984	0.2952	NSCLC - 6 months
at 6m - il2	at 6m - il10	10	0.11	-0.56	0.69	0.7635	0.7635	NSCLC - 6 months
at baseline - tnfa	at baseline - il2	15	0.49	-0.03	0.80	0.0631	0.0946	Melanoma - Baseline
at baseline - tnfa	at baseline - il10	15	0.66	0.23	0.88	0.0068	0.0205	Melanoma - Baseline
at baseline - il2	at baseline - il10	15	0.29	-0.26	0.70	0.2983	0.2983	Melanoma - Baseline
at 3m - tnfa	at 3m - il2	7	0.71	-0.08	0.95	0.0713	0.0713	Melanoma - 3 months
at 3m - tnfa	at 3m - il10	7	0.93	0.58	0.99	0.0025	0.0076	Melanoma - 3 months
at 3m - il2	at 3m - il10	7	0.79	0.08	0.97	0.0362	0.0544	Melanoma - 3 months
at 6m - tnfa	at 6m - il2	5	0.60	-0.60	0.97	0.2848	0.2848	Melanoma - 6 months
at 6m - tnfa	at 6m - il10	5	0.70	-0.48	0.98	0.1881	0.2822	Melanoma - 6 months
at 6m - il2	at 6m - il10	5	0.90	0.09	0.99	0.0374	0.1122	Melanoma - 6 months
