# Supplementary figures and images for: Serum TNF -α, IL-10 and IL-2 Trajectories and Outcomes in NSCLC and Melanoma Under Anti-PD-1 Therapy: Longitudinal Real-World Evidence from a Single Center
Source: Curr Issues Mol Biol. 2025 Sep 11;47(9):746. doi: 10.3390/cimb47090746 (PMC12468888; doi:10.3390/cimb47090746)

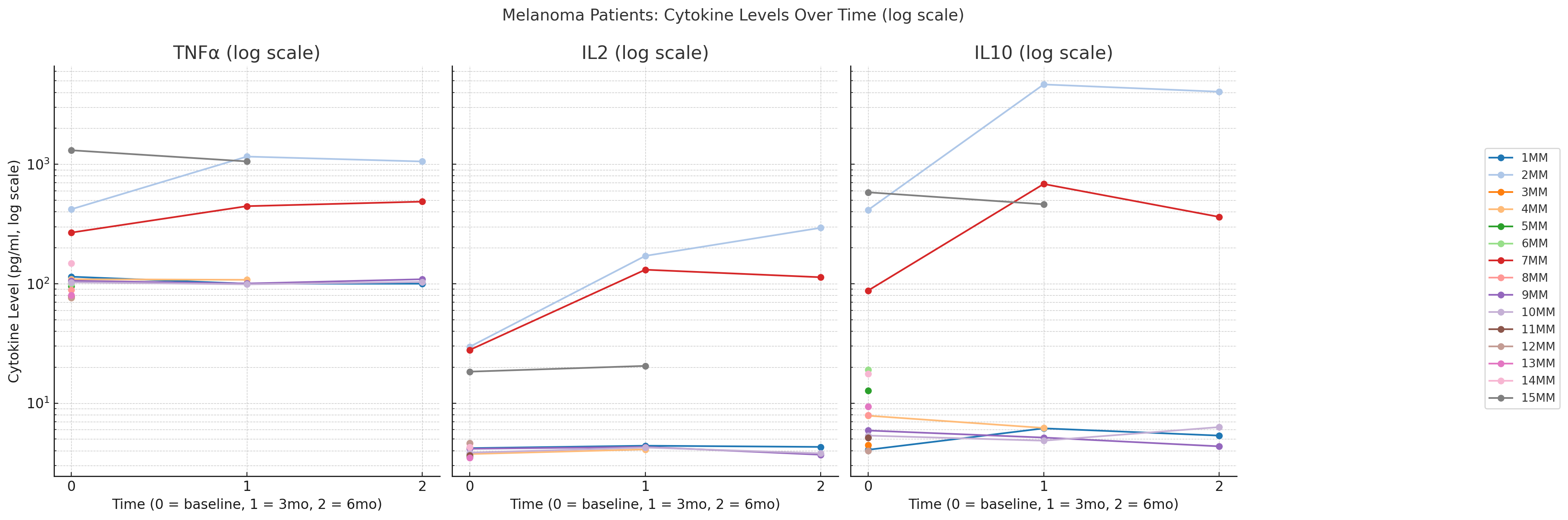

Supplement: Supplementary file 1 [file cimb-47-00746-s001.zip › Supplementary Materials-ALL MM- outliers included.png]

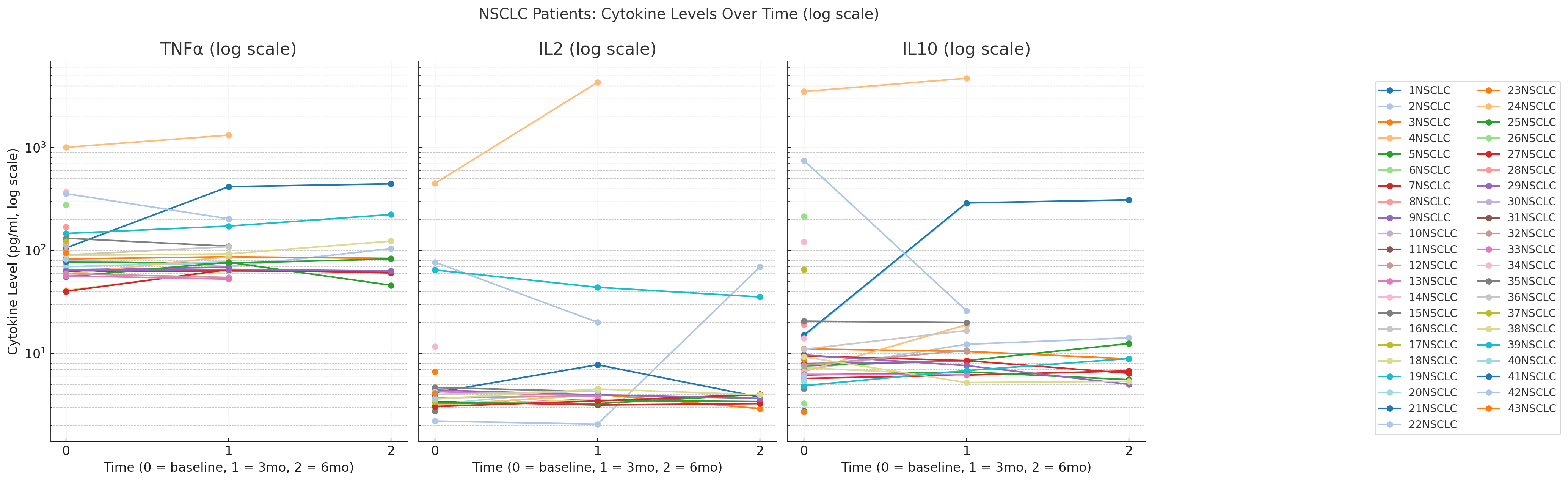

Supplement: Supplementary file 1 [file cimb-47-00746-s001.zip › Supplementary Materials-ALL NSCLC - outliers included.png]

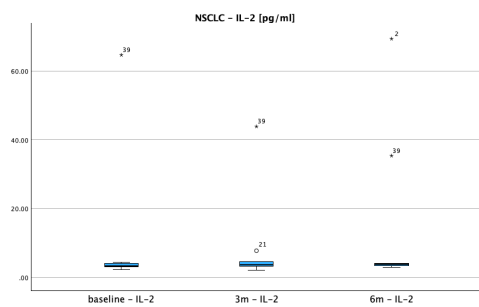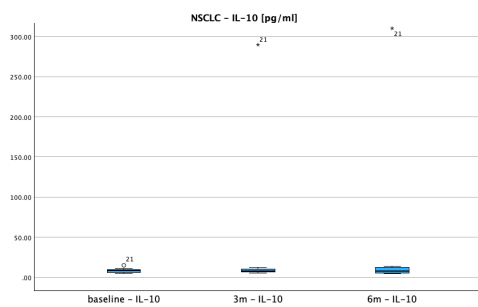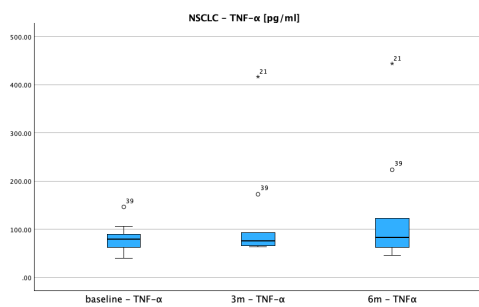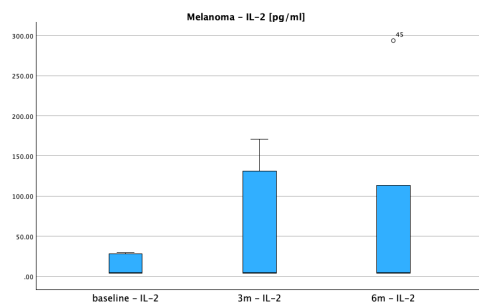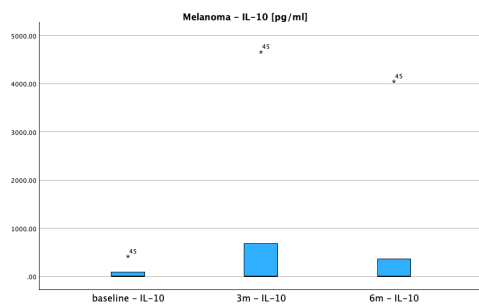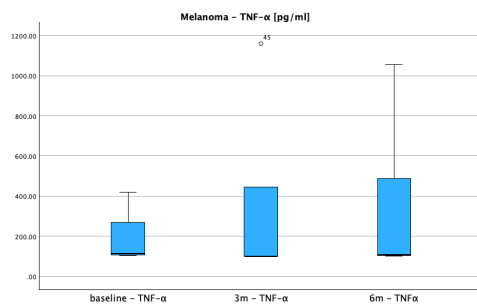

Supplement: Supplementary file 1 [file cimb-47-00746-s001.zip › Supplementary Materials-boxplot.pdf]

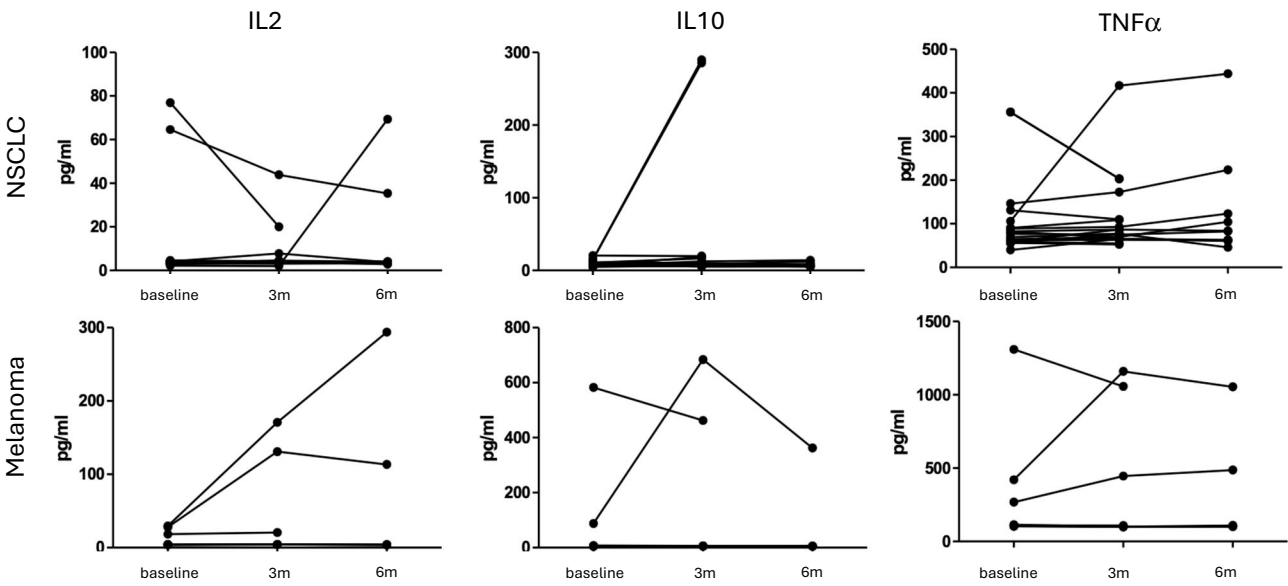

Supplement: Supplementary file 1 [file cimb-47-00746-s001.zip › Supplementary Materials-Longitudinal changes in serum cytokine levels.pdf]
